# Skeletal Muscle 11beta-HSD1 Controls Glucocorticoid-Induced Proteolysis and Expression of E3 Ubiquitin Ligases Atrogin-1 and MuRF-1

**DOI:** 10.1371/journal.pone.0016674

**Published:** 2011-01-31

**Authors:** Katrin Biedasek, Janin Andres, Knut Mai, Stephanie Adams, Simone Spuler, Jens Fielitz, Joachim Spranger

**Affiliations:** 1 Department of Endocrinology, Diabetes and Nutrition, Charité-Universitätsmedizin Berlin, Berlin, Germany; 2 Muscle Research Unit, Experimental and Clinical Research Center, Charité-Universitätsmedizin Berlin, Berlin, Germany; 3 Department of Cardiology and Experimental and Clinical Research Center, Charité-Universitätsmedizin Berlin, Berlin, Germany; Paris Institute of Technology for Life, Food and Environmental Sciences, France

## Abstract

Recent studies demonstrated expression and activity of the intracellular cortisone-cortisol shuttle 11beta-hydroxysteroid dehydrogenase type 1 (11beta-HSD1) in skeletal muscle and inhibition of 11beta-HSD1 in muscle cells improved insulin sensitivity. Glucocorticoids induce muscle atrophy via increased expression of the E3 ubiquitin ligases Atrogin-1 (Muscle Atrophy F-box (MAFbx)) and MuRF-1 (Muscle RING-Finger-1). We hypothesized that 11beta-HSD1 controls glucocorticoid-induced expression of atrophy E3 ubiquitin ligases in skeletal muscle. Primary human myoblasts were generated from healthy volunteers. 11beta-HSD1-dependent protein degradation was analyzed by [^3^H]-tyrosine release assay. RT-PCR was used to determine mRNA expression of E3 ubiquitin ligases and 11beta-HSD1 activity was measured by conversion of radioactively labeled [^3^H]-cortisone to [^3^H]-cortisol separated by thin-layer chromatography. We here demonstrate that 11beta-HSD1 is expressed and biologically active in interconverting cortisone to active cortisol in murine skeletal muscle cells (C2C12) as well as in primary human myotubes. 11beta-HSD1 expression increased during differentiation from myoblasts to mature myotubes (p<0.01), suggesting a role of 11beta-HSD1 in skeletal muscle growth and differentiation. Treatment with cortisone increased protein degradation by about 20% (p<0.001), which was paralleled by an elevation of Atrogin-1 and MuRF-1 mRNA expression (p<0.01, respectively). Notably, pre-treatment with the 11beta-HSD1 inhibitor carbenoxolone (Cbx) completely abolished the effect of cortisone on protein degradation as well as on Atrogin-1 and MuRF-1 expression. In summary, our data suggest that 11beta-HSD1 controls glucocorticoid-induced protein degradation in human and murine skeletal muscle via regulation of the E3 ubiquitin ligases Atrogin-1 and MuRF-1.

## Introduction

Glucocorticoid excess is associated with central obesity, insulin resistance, arterial hypertension and skeletal muscle atrophy. Intracellular glucocorticoid signaling is pre-receptor-controlled by 11beta-hydroxysteroid dehydrogenase type 1 (11beta-HSD1) which activates cortisol from the hormonally inactive cortisone. Increased 11beta-HSD1 activity has been associated with symptoms of the metabolic syndrome. Although obese individuals have normal cortisol plasma levels, intracellular glucocorticoid action in liver and adipose tissue were shown to be increased in some publications due to high 11beta-HSD1 expression levels and activity [1], [2], [3]. Transgenic mice over-expressing 11beta-HSD1 specifically in adipose tissue gained weight and developed insulin resistance and dyslipidemia [4]. In contrast, 11beta-HSD1 knockout mice were resistant to obesity and hyperglycemia under a high-fat diet [5].

In adipose tissue and liver, the function of 11beta-HSD1 is well studied but data about its role in skeletal muscle are sparse although it is well known that glucocorticoids induce muscle atrophy by inhibition of protein synthesis and induction of protein degradation [6], [7]. Very recently, expression and activity of 11beta-HSD1 was shown to be increased in skeletal muscle of diabetic individuals [8] and pharmacological inhibition of 11beta-HSD1 reversed cortisone-disturbed insulin signaling in skeletal muscle cells [9]. 11beta-HSD1 was also suggested to be involved in the differentiation of skeletal myoblasts to mature myotubes due to an increasing 11beta-HSD1 expression during the differentiation process, shown in the skeletal muscle C2C12 cell line [10], [11]. Despite those convincing data that 11beta-HSD1 is functionally active in skeletal muscle, the underlying role of this enzyme for muscle atrophy associated pathways is still unclear. Glucocorticoids are well established to induce skeletal muscle proteolysis primarily by activating the ubiquitin-proteasome-system (UPS) and increasing the expression of the two E3 ubiquitin ligases Atrogin-1 and MuRF-1 [12], [13], [14], [15].

We therefore analyzed in the skeletal muscle cell line C2C12 and in primary human myotubes whether 11beta-HSD1 controls glucocorticoid-induced protein degradation and whether this effect is attributed to an increased expression of the E3 ubiquitin ligases Atrogin-1 and MuRF-1.

## Materials and Methods

### Cell Culture

The C2C12 murine myoblast cell line and primary human myoblasts were grown under standard conditions. For C2C12, growth medium consisted of Dulbecco's modified Eagle's medium (DMEM) including 4.5 g/L glucose and stable glutamine, supplemented with 10% fetal calf serum (FCS). Primary human myoblast cultures were isolated by protease digestion from fresh muscle biopsies (collagenase II, dispase1, trypsin/EDTA) and expanded in skeletal muscle growth medium including supplement mix (PromoCell, Heidelberg, Germany), 10% FCS, glutamine (3 mM) and gentamycin (40 µg/ml). All cultures were enriched in myoblasts by immuno-magnetic cell sorting using anti-CD56/NCAM antibody coated magnetic beads (Miltenyi Biotech, Bergisch Gladbach, Germany). Purity of the myoblast preparation was checked by staining with an anti-desmin antibody revealing more than 95% desmin-positive cells. All experiments were performed between passage P5 and P15 after isolation to avoid premature replicative senescence. Differentiation of myoblasts into myotubes was initiated at approximately 90% confluence by switching to differentiation medium containing 2% horse serum (D0, day 0 of differentiation). For stimulation experiments, myotubes were serum-starved and on day six of differentiation (D6) 30–60 min pre-treated with 10 µM carbenoxolone (Sigma-Aldrich Chemie GmbH, Munich, Germany), followed by incubation with 1 µM cortisone (Sigma-Aldrich Chemie GmbH, Munich, Germany) and 1 µM dexamethasone (Sigma-Aldrich Chemie GmbH, Munich, Germany) for sixteen hours.

### Real-time PCR

Total RNA was isolated using a commercial RNA isolation kit (Roche Diagnostics GmbH, Mannheim, Germany). cDNA was also synthesized according to manufacturer's protocol (Applied Biosystems, Darmstadt, Germany). Real-time PCR (RT-PCR) was analyzed using Power Sybr Green (Applied Biosystems, Darmstadt, Germany) on a 7300 Real-Time PCR System (Applied Biosystems, Darmstadt, Germany) or by TaqMan Technology (Applied Biosystems, Darmstadt, Germany). All experiments were performed at least in triplicate. The PCR primer sequences will be provided upon request.

### 11beta-HSD1 activity assay

11beta-HSD1 activity was measured as described previously [16]. Briefly, we determined the conversion of radioactively labeled [^3^H]-cortisone to [^3^H]-cortisol. Cells were incubated with 0.1 µCi 1,2-[^3^H]-Cortisone (American Radiolabeled Chemicals, Inc., St. Louis, USA) at 37°C and 5% CO_2_ for at least four hours in serum-free DMEM. Then, medium supernatant was collected and cells were lysed with 50 mM NaOH. An aliquot of cell lysate was used to determine DNA concentration in a photometer at 260 nm. Supernatant and cell lysate were mixed with 2–3 volumes of ethyl-acetate (Merck, Darmstadt, Germany) by shaking and lower hydrophil and upper lipophil phases were separated by centrifugation. The upper steroid containing phase was fully evaporated under air, resolved in dichlormethane (Carl Roth GmbH + Co. KG, Karlsruhe, Germany) and cortisone (E) and cortisol (F) separated by thin-layer chromatography using dichlormethane:methanol (75:5) as solvent. [^3^H]-labeled cortisone and cortisol were quantified in a beta-counter.

### Protein degradation

Proteolysis was measured by determining the rate of release of trichloroacetic acid (TCA)-soluble proteins radioactively labeled with [^3^H]-tyrosine into the media. On day five of differentiation, cells were pre-labeled with 0.5 µCi L-[3,5-^3^H]-tyrosine/ml (Biotrend GmbH, Köln, Germany) at 37°C and 5% CO_2_ for two days in differentiation medium. The cells were washed once with hank's buffered salt solution (HBSS) and transferred to non-radioactive serum-free DMEM containing 2 mM tyrosine for two hours to exclude proteolysis of short-lived proteins. Then the cells were washed twice with HBSS and again transferred to non-radioactive serum-free DMEM containing 2 mM tyrosine. Release of [^3^H]-tyrosine was measured after pre-treatment with 10 µM carbenoxolone for 30–60 min followed by stimulation with 1 µM cortisone and 1 µM dexamethasone. After 16 hours incubation, culture medium was transferred to a microcentrifuge tube containing 100 µl bovine serum albumin (BSA) (10 mg/ml). For precipitation, an equal volume of TCA (20% wt/vol) was added to a final concentration of 10% (wt/vol) and incubated at 4°C for at least one hour until overnight. Samples were then centrifuged at 14000 g for 5 min. The supernatant contained the TCA-soluble proteins (fraction A). The TCA-insoluble proteins in the precipitate were solubilized in 0.5 M NaOH und 0.1% Triton X100 (fraction B). The cell monolayer was washed twice with phosphate buffered saline (PBS) and also solubilized in 0.5 M NaOH and 0.1% Triton X100 (fraction C). [^3^H]-labeled tyrosine within the three fractions was quantified using a beta-counter. Percentage protein degradation was calculated as 100×[fraction A/(fraction A+B+C)].

### Statistics

Results are presented as means ± SEM. Mann-Whitney-U test was used to analyze differences between groups. Significance was considered for p<0.05. All experiments were performed at least in triplicate.

## Results

### 11beta-HSD1 expression increases during myoblast differentiation

**Figure 1:**
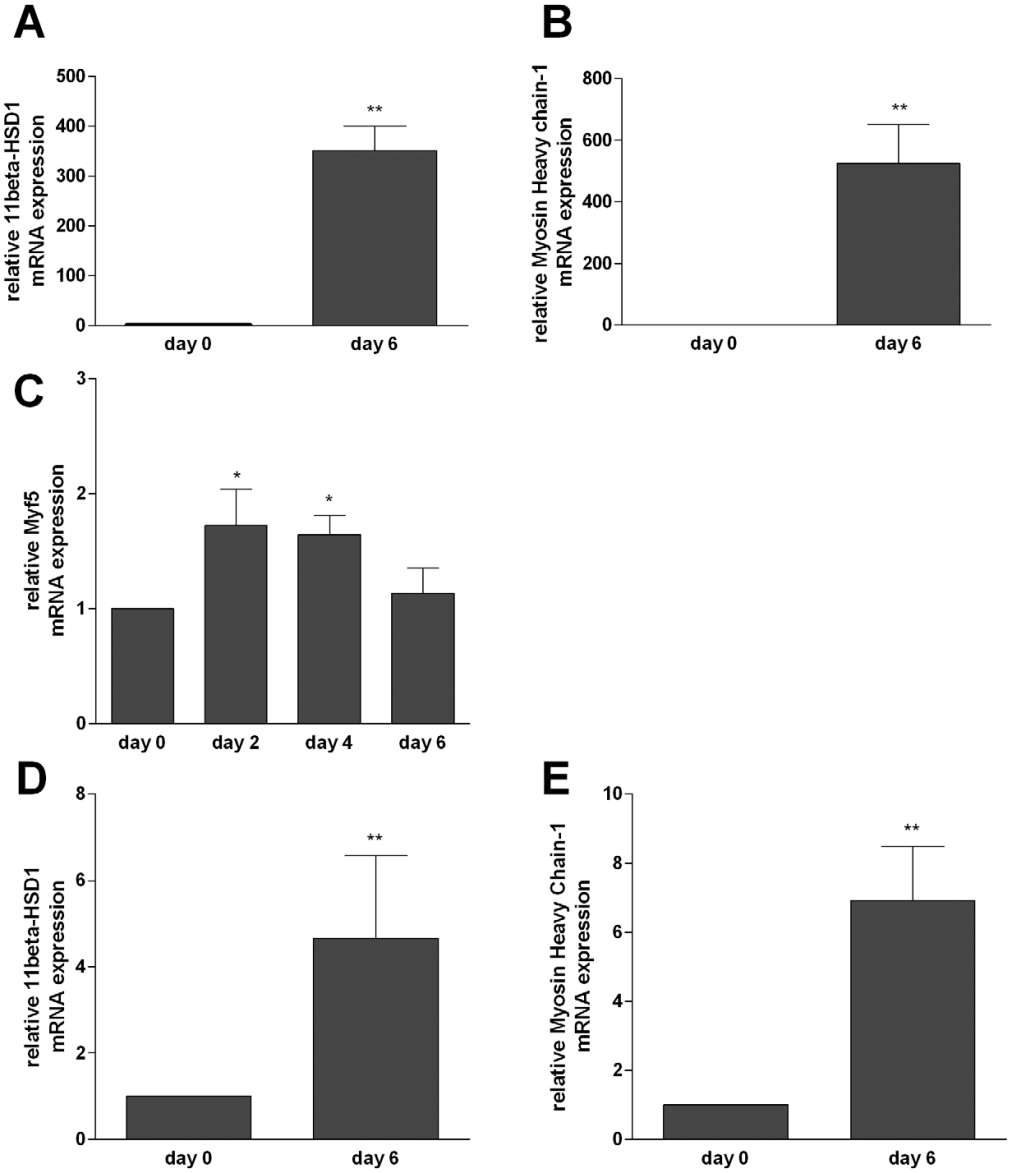
Regulation of 11beta-HSD1 and differentiation markers during differentiation of C2C12 cells and primary human myoblasts. Data were normalized to mRNA expression of 16s-RiboProtein. Mean ± SEM of at least three experiments are shown. Relative 11beta-HSD1 mRNA expression in C2C12 (**A**). Relative Myosin Heavy Chain-1 mRNA expression in C2C12 cells (**B**). Relative Myf5 mRNA expression in C2C12 cells (**C**). Relative 11beta-HSD1 mRNA expression in primary human myoblasts (**D**). Relative Myosin Heavy Chain-1 mRNA expression in primary human myoblasts (**E**). * p<0.05, ** p<0.01, *** p<0.0001.

To test whether 11beta-HSD1 expression depends on the differentiation process of skeletal muscle cells from myoblasts to adult myotubes, we measured mRNA expression of 11beta-HSD1 and the differentiation markers Myosin Heavy chain-1 and Myf5 at different time points (day 0 (D0), day 2 (D2), day 4 (D4) and day (D6)). Expression of Myosin Heavy chain-1 (marker of terminal differentiation) strongly increased from D0 to D6 (Fig. 1B and 1E) confirming differentiation into adult myotubes. Additionally, Myf5 mRNA expression (marker of myoblast fusion) reached a plateau on D2 (Fig. 1C). 11beta-HSD1 mRNA expression was low in myoblasts (D0) and increased at D6 by factor 351.2±48.6 (p<0.01) in C2C12 cells (Fig. 1A) and 4.7±1.9 (p<0.01) in primary human myotubes (Fig. 1D), whereas 11beta-HSD1 was already higher expressed in undifferentiated primary human myoblasts than in C2C12 myoblasts. In summary, we demonstrate that 11beta-HSD1 mRNA expression is induced within the differentiation process of the murine C2C12 cell line, but also in primary human myoblasts.

### 11beta-HSD1 controls glucocorticoid-induced proteolysis and expression of the E3 ubiquitin ligases Atrogin-1 and MuRF-1

**Figure 2:**
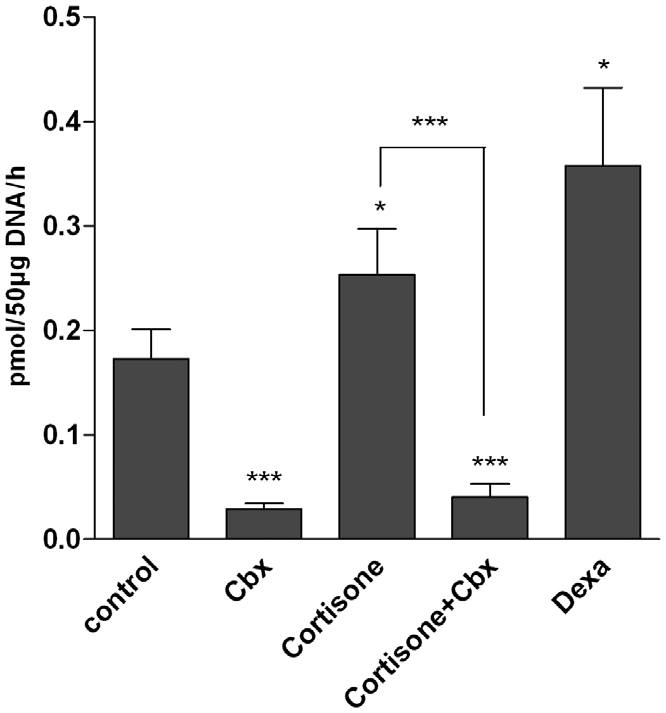
Effects of cortisone, dexamethasone (dexa) and inhibition of 11beta-HSD1 by carbenoxolone (Cbx) on 11beta-HSD1 activity in C2C12. Mean ± SEM of at least three experiments are shown. * p<0.05, ** p<0.01, *** p<0.0001.

**Figure 3:**
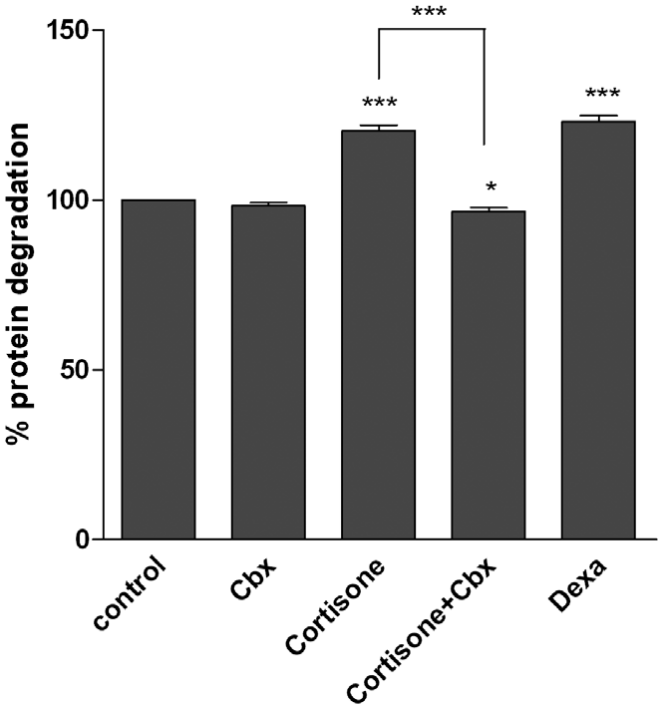
Effects of cortisone, dexamethasone (dexa) and inhibition of 11beta-HSD1 by carbenoxolone (Cbx) on protein degradation in C2C12 myotubes. Protein degradation was measured by determining the rate of release of TCA-soluble proteins radioactively labeled with [^3^H]-tyrosine into the media. Mean ± SEM of at least three experiments are shown. * p<0.05, ** p<0.01, *** p<0.0001.

**Figure 4:**
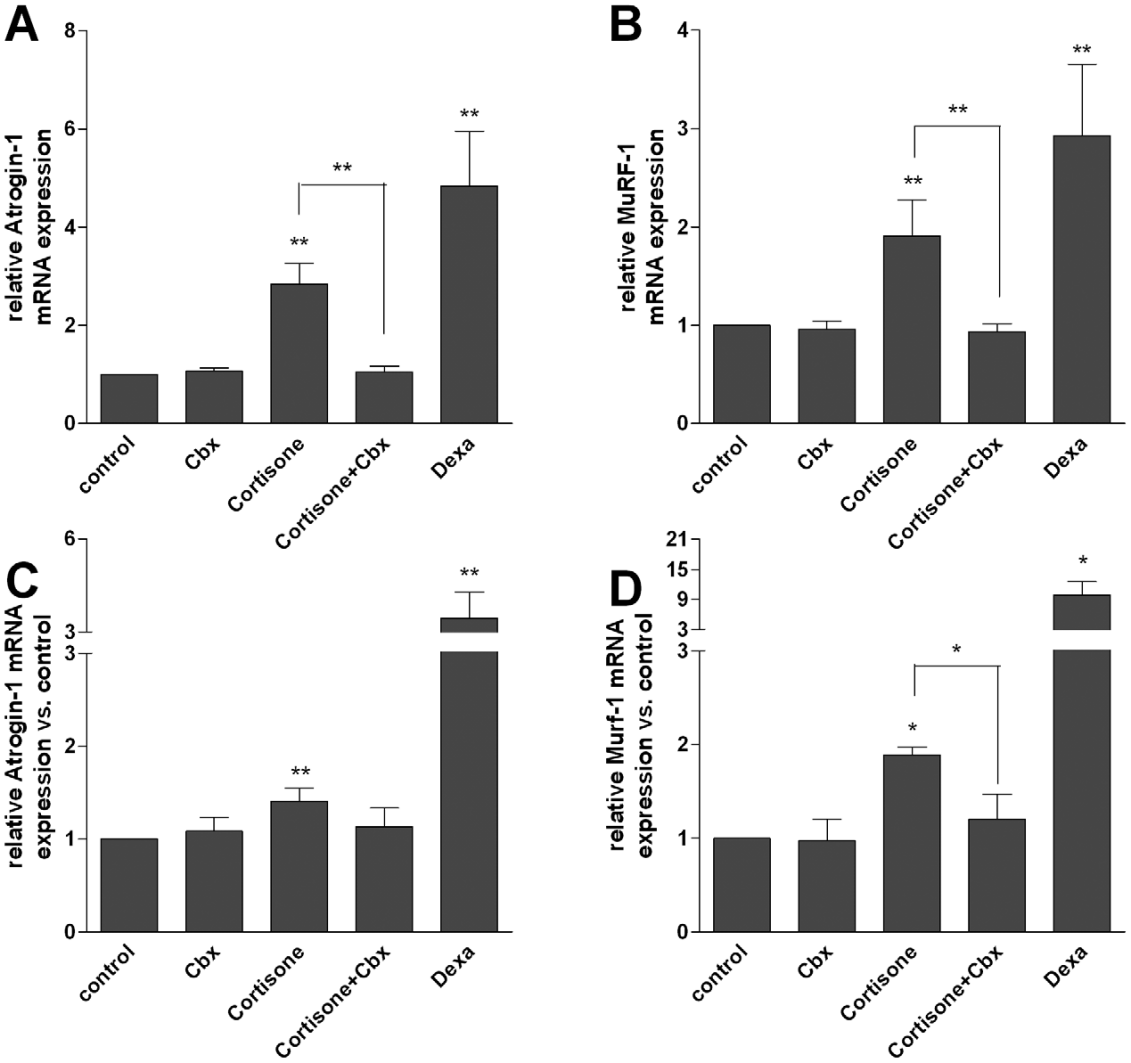
Effects of cortisone, dexamethasone (dexa) and inhibition of 11beta-HSD1 by carbenoxolone (Cbx) on Atrogin-1 and MuRF-1 expression. Relative Atrogin-1 and MuRF-1 mRNA expression in C2C12 (**A and B**) and in primary human myotubes (**C and D**). Data are normalized to mRNA expression of 16s-RiboProtein. Mean ± SEM of at least three experiments are shown. * p<0.05, ** p<0.01, *** p<0.0001.

To analyze the role of 11beta-HSD1 on skeletal muscle proteolysis, we induced 11beta-HSD1 activity by stimulation with cortisone and dexamethasone (positive control) and inhibited it by carbenoxolone (Fig. 2). We observed an increased proteolysis in cortisone-treated C2C12 myotubes (E 120.3±1.7% vs. control, p<0.001) (Fig. 3). This was paralleled by a cortisone-dependent increase of the E3 ubiquitin ligases Atrogin-1 (2.84±0.4 vs. control, p<0.01) (Fig. 4A) and MuRF-1 (1.91±0.4 vs. control, p<0.01) (Fig. 4B). We next analyzed the role of 11beta-HSD1 in the regulation of protein degradation and those E3 ubiquitin ligases. Indeed, the cortisone-induced increase of protein degradation was completely abolished by pre-inhibition of 11beta-HSD1 with carbenoxolone (E+Cbx 96.5±1.3% vs. control, p<0.001) (Fig. 3). Comparably, inhibition of 11beta-HSD1 completely abolished the effect of cortisone on Atrogin-1 (E+Cbx 1.05±0.1 vs. control, p<0.01) and MuRF-1 (E+Cbx 0.94±0.1 vs. control, p<0.01) (Fig. 4A and 4B). These results were basically confirmed in primary human myotubes (MuRF-1: E+Cbx 1.2±0.3 vs. E 1.9±0.1, p<0.05), although slightly failing significance for Atrogin-1 (1.1±0.2 vs. E 1.4±0.1, p = 0.3) (Fig.4C and 4D).

## Discussion

Here we demonstrate that 11beta-HSD1 controls glucocorticoid-induced protein degradation and transcription of the E3 ubiquitin ligases Atrogin-1 and MuRF-1 in the skeletal muscle cell line C2C12 and in primary human myotubes. In addition, we show that 11beta-HSD1 mRNA expression increases during differentiation of primary human myoblasts to mature myotubes indicating a role of 11betaHSD1 in differentiation of myocytes.

Atrogin-1 and MuRF-1 are upregulated in various models of muscle atrophy and are considered to serve as reliable markers of muscular atrophy [17], [18], [19]. An increased expression of Atogin-1 and MuRF-1 strongly indicates an enhanced proteolysis via the UPS, and several studies demonstrated that glucocorticoids induce muscle proteolysis via Atrogin-1 and MuRF-1 [12], [13], [14], [17]. This is supported by our results showing glucocorticoid-induced increases of proteolysis and atrophy signaling. In addition to existing data our results demonstrate that glucocorticoid-dependent effects are partially pre-receptor controlled by 11beta-HSD1. A limitation of our study is the use of carbenoxolone as a pharmacological 11beta-HSD1 inhibitor. Although carbenoxolone is an established inhibitor of 11beta-HSD1 and we achieved a near-complete inhibition of 11beta-HSD1 activity in our experiments, we cannot entirely exclude unspecific effects. Clearly, the confirmation of our results in animal experiments e.g. using mice with myocyte-specific 11beta-HSD1 knock-out would be highly desirable. Vice versa, a specific strength of our study is the confirmation of those results in human myotubes, pointing towards a physiological relevance of the findings in humans.

Previous studies suggested 11beta-HSD1 mRNA expression in skeletal muscle [8], [20], [21] and recent data supported that 11beta-HSD1 controls metabolic phenotypes in skeletal muscle such as insulin sensitivity. Morgan *et al.* (2009) showed that pharmacological inhibition of 11beta-HSD1 in mice reversed cortisone-disturbed insulin signaling pathway in skeletal muscle cells by blocking amongst others the decrease of Akt/PKB phosphorylation [9]. The PI3K/Akt pathway is also involved in the proteolytic signaling cascade in skeletal muscle. Thus, the activation of the PI3K/Akt pathway is diminished in myotubes undergoing atrophy [22]. Accordingly, glucocorticoids decrease Akt/PKB phosphorylation followed by an activation of the Forkhead box O (Foxo) class transcription factors causing activation of the Atrogin-1 promoter and a decrease in muscle fiber size [23]. Our data strongly support that atrophy signaling in skeletal muscle also depends on pre-receptor controlled mechanisms. Theoretically, the observed effects on atrophy signaling may depend on the associated metabolic consequences or vice versa, although this is unlikely given the setting of our studies which were performed without insulin stimulation. Nevertheless, this question was not directly addressed and we cannot exclude that a modification of basal insulin sensitivity may link glucocorticoids, 11beta-HSD1 and atrophy signaling.

In conclusion, our data suggest that 11beta-HSD1 controls glucocorticoid-induced protein degradation in skeletal muscle by regulating the expression of the muscle ubiquitin E3 ligases Atrogin-1 and MuRF-1.
